# Hypertension-Induced Biomechanical Modifications in the Aortic Wall and Their Role in Stanford Type B Aortic Dissection

**DOI:** 10.3390/biomedicines12102246

**Published:** 2024-10-02

**Authors:** Yuhao Wei, Da Li, Chengxin Weng, Jiarong Wang, Ding Yuan, Tinghui Zheng

**Affiliations:** 1 Department of Mechanics & Engineering, College of Architecture & Environment, Sichuan University, Chengdu 610065, China; weiyuhaoo@stu.scu.edu.cn; 2 Yibin Institute of Industrial Technology, Sichuan University Yibin Park, Yibin 644000, China; 3 Department of Engineering Mechanics, School of Ocean and Civil Engineering, Shanghai Jiao Tong University, Shanghai 200240, China; da_li@sjtu.edu.cn; 4 Division of Vascular Surgery, Department of General Surgery, West China Hospital, Sichuan University, Chengdu 610041, China; chengxin_weng@wchscu.cn (C.W.); wangjiarong@wchscu.cn (J.W.); 5 Med-X Center for Informatics, Sichuan University, Chengdu 610041, China

**Keywords:** stable hypertension, unstable hypertension, biomechanical property, Stanford type B aortic dissection, blood pressure management

## Abstract

Objective: Hypertension is a major risk factor for the type B aortic dissection (TBAD), while many patients do not manage or regulate their hypertension consistently, leading to stable or unstable hypertension. Currently, the effects of stable and unstable hypertension on the biomechanical properties of the aorta remain unclear. The objective was to identify a blood pressure state that represents a greater risk for TBAD development. Methods: A total of 183 samples (108 axial and 75 circumferential) were divided into three groups. Fatigue tensile tests were carried out to simulate normotension, stable hypertension, and unstable hypertension conditions, respectively. Uniaxial tensile tests were performed; thus, the elastic modulus, energy loss, and the peeling force were assessed to evaluate the biomechanical properties. Results: Compared with normal blood pressure, the modulus of elastic fibers decreased under stable hypertension (0.05 ± 0.02 MPa vs. 0.11 ± 0.03 MPa, *p* < 0.001) and unstable hypertension (0.08 ± 0.02 MPa, *p* = 0.008), while collagen fibers increased under stable hypertension (2.14 ± 0.51 MPa vs. 1.10 ± 0.24 MPa, *p* < 0.001) but decreased under unstable hypertension (0.52 ± 0.14 MPa, *p* < 0.001) in the axial direction. Similar trends were observed circumferentially. Energy loss was highest under unstable hypertension (0.16 ± 0.03 vs. 0.08 ± 0.03, *p* < 0.001). Peeling force was significantly reduced under stable hypertension (81.69 ± 12.72 N/m vs. 111.10 ± 27.65 N/m, *p* < 0.001) and further under unstable hypertension (71.37 ± 16.13 N/m, *p* < 0.001). Conclusions: Stable and unstable hypertension significantly impair the biomechanical properties of the aortic wall, with unstable hypertension leading to greater damage. Hypertensive patients are recommended to strictly follow medical advice to control blood pressure to avoid a higher risk of TBAD due to improper blood pressure management.

## 1. Introduction

Aortic dissection (AD) is a critical cardiovascular condition characterized by the rupture of the intima–media of the aortic wall. This rupture allows blood to enter the torn aortic wall, leading to separation of the intima–media and the formation of a dissection. AD can result in aortic regurgitation, distal malperfusion, and even aortic rupture [1]. According to the classic Stanford classification system, a dissection that does not involve the ascending aorta is classified as Stanford type B aortic dissection (TBAD). Notably, the short-term mortality rate in patients with undetected or untreated acute TBAD has been reported to exceed 90% [2].

Biomechanical indices play a crucial role in assessing the risk of developing aortic diseases, including TBAD, with abnormal indices usually indicating a higher risk of AD development [3]. Guo et al. conducted biaxial tensile tests on ascending aortic specimens from both healthy individuals and those with type A aortic dissection (TAAD) and revealed a significantly lower elastic modulus in TAAD specimens compared to healthy samples [4]. In a recent study, the mechanical properties of specimens from TAAD patients who underwent ascending aortic replacement were examined, and their results showed significant changes in both the elastic potential energy and the energy loss at the initial rupture, indicating a reduction in elasticity and compliance [5]. This suggests that the biomechanical properties of the aortic wall around the initial tear may undergo alterations before AD occurs, with poorer biomechanical properties potentially predisposing to AD. Kurihara et al. found that the peeling force of descending aortic samples from rats with TBAD was significantly lower than that of normal rat samples, even before TBAD onset [6]. These findings suggest that biomechanical indices such as elastic modulus, energy loss, and peeling force can effectively differentiate AD and may even predict its onset and progression.

Hypertension is a major predisposing factor for the development of TBAD, particularly in Asian populations, and the prevalence of acute TBAD in individuals with hypertension has been reported to be 80.9% [7,8,9]. Research by Wang et al. indicated that the overall prevalence of hypertension in China is 28.9%, with only 13.4% of hypertensive patients adopting blood pressure control measures [10]. Unfortunately, many hypertensive patients do not consistently manage their blood pressure. In a study by Dong et al., 28.6% of patients who attempted to control their blood pressure did not do so regularly, and 56.3% of these patients developed acute aortic dissection (AAD) [11]. These findings suggest that both uncontrolled, sustained stable hypertension and unstable hypertension induced by poor blood pressure management may be high-risk factors for AAD, while poorer medication compliance is a significant trigger for the occurrence of AAD, posing a substantial challenge to long-term health. However, despite the growing consensus on blood pressure management, its impact on the prognosis of patients with TBAD remains unclear [12]. Furthermore, the influence of different hypertension states on the biomechanical properties of the aortic wall still requires further understanding. Therefore, it is necessary to investigate how these biomechanical properties change under various hypertension conditions to identify which conditions lead to poorer biomechanical performance of the aorta, thereby further reducing the risk of TBAD occurrence.

In existing studies, healthy aortic tissue is typically sourced from cadavers, making it a significant challenge to collect a sufficient number of aortic samples [4,13,14]. Due to the challenges in obtaining healthy and fresh human aorta specimens, the porcine aorta is a commonly employed substitute due to its histological similarity to the human aorta and its biomechanical properties that closely resemble those of human aortic tissues under 60 years of age [15]. Consequently, the porcine aorta is extensively utilized in biomechanical studies aimed at evaluating aortic disorders [3].

Currently, the effects of stable and unstable hypertension on the biomechanical properties of the aorta still remain unclear. Therefore, the aim of this study was to examine the impact of stable hypertension and unstable hypertension on the biomechanical characteristics of the porcine aortic wall. Based on the uniaxial tensile experimental approach, we investigated alterations in the biomechanical properties of the aortic wall under both stable and unstable hypertension conditions. Our objective was to identify which blood pressure state represents a greater risk for TBAD development. We hope this study will offer valuable insights for future clinical blood pressure management.

## 2. Materials and Methods

### 2.1. Porcine Aortic Specimen Preparation

This study was approved by the Ethics Committee on Biomedical Research of West China Hospital, Sichuan University (protocol code: 20240522001).

**Figure 1:**
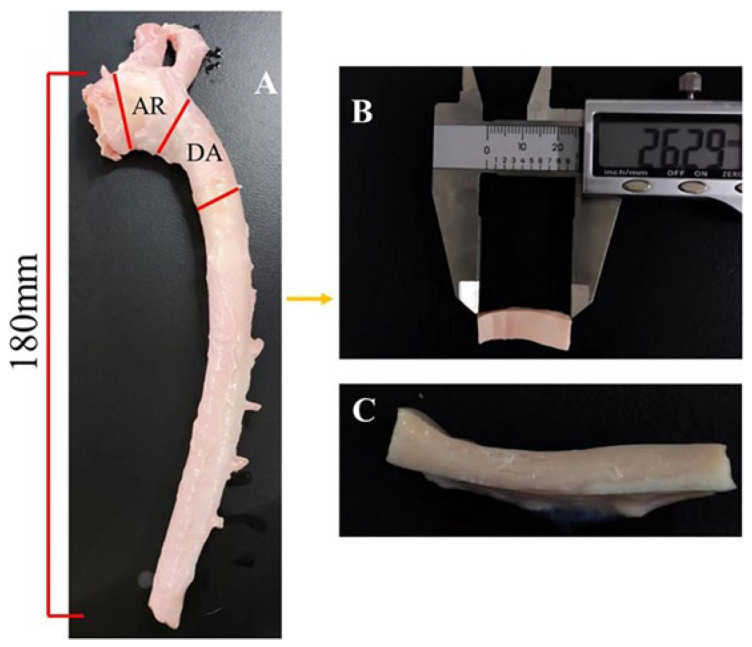
Schematic diagram of porcine aortic wall specimen preparation. AR, aortic arch; DA, descending aorta. (**A**) the aorta collected for the aortic wall sample preparation; (**B**) aortic wall sample used for further mechanical test; (**C**) cross-section of aortic wall sample.

The porcine aortic samples used in this study were sourced from native pigs in Sichuan, China. All aortas were harvested from healthy piglets weighing approximately 120 kg. Following dissection, the porcine aortas were promptly immersed in sterile phosphate-buffered saline and transported to the biomechanics laboratory within 2 h at a temperature of 4 °C. A total of 10 fresh porcine aorta samples were collected for the study (with the age being 21.5 ± 1.4 weeks). Before the experiment, tissues such as grease and fascia that surrounded the exterior of the aorta were removed to minimize their impact on the aortic wall, as shown in Figure 1A.

It has been reported that the biomechanical properties of the porcine aortic wall exhibit gradual changes along the axial direction [16]. Therefore, only the TBAD-susceptible part (from the cardiac outlet to approximately 18 cm downstream along the aorta) was selected for the following experiments. The aortas were sectioned into rectangular samples, as illustrated in Figure 1B. Eventually, a total of 183 samples (108 axial and 75 circumferential) were obtained. The samples were measured at 25.17 ± 2.31 mm in length, 10.18 ± 1.74 mm in width, and 2.24 ± 0.17 mm in thickness.

### 2.2. Uniaxial Tensile Experiment

Compared to biaxial tensile testing, uniaxial tensile testing is more helpful for accurately understanding the stress–strain curve and ultimate failure strength of aortas, which is more suited for destructive experiments. Currently, uniaxial tensile testing is widely used in determining the biomechanical properties of aortas [3]. By employing a quasi-static loading method, the mechanical properties of the aorta could be easily obtained.

Amabili, M. et al. tested the dynamic elastic modulus of the aorta at different stretching frequencies, and their results revealed that, compared to the static elastic modulus, the dynamic elastic modulus of the aorta significantly increased at a stretching frequency of 1 Hz. However, as the stretching frequency increased further, the changes in the dynamic elastic modulus became less significant, suggesting that a stretching frequency of 1 Hz effectively replicates the dynamic mechanical behavior of the aorta [17]. Furthermore, a stretching frequency of 1 Hz typically corresponds to the physiological condition of a heart rate of 60 bpm. Therefore, fatigue experiments in the current study were carried out at a frequency of 1 Hz over a total period of 7200 s.

The hypertension state was simulated by the variations in strain rates. It has been reported that the average strain of healthy aortas under normal blood pressure is generally less than 10% [18,19]. Under the hypertension condition, the load on the healthy aortic wall increases and therefore leads to an increase in strain. The study by Zhu et al. has indicated that the difference in aortic diameter between systole and diastole in hypertensive patients have exceeded 20% [20]. Since hypertensive patients typically have reduced aortic elasticity, the strain of a healthy aorta under hypertensive load should be even greater. Thus, strain rates at 10% and 30% were set to simulate normal blood pressure and hypertensive conditions, respectively.

All mechanical tests in this study were conducted using an IBTC-300 uniaxial mechanical testing machine (IBTC-300, Care Measurement & Control Co., Ltd., Tianjin, China). To establish a stable displacement–load relationship, 10 quasi-static stretches at 5% strain were performed before uniaxial tensile testing. All tensile experiments were conducted at room temperature and completed within 12 h of specimen collection.

In total, this study performed stable hypertension and unstable hypertension fatigue stretching experiments, and a normal blood pressure state was also simulated as a control. The detailed experimental procedures are presented in Supplementary Methods (Supplementary Materials).

### 2.3. Biomechanical Parameters of the Aortic Wall

#### 2.3.1. Elastic Modulus

To investigate the changes in the elastic modulus of elastic and collagen fibers, a mathematical model that combines both elastic and collagen fibers was introduced, as proposed by Raghavan et al. [21], with the following expression:

(1) $$\varepsilon =\lambda -1=\left(K+\frac{A}{B+\sigma }\right)\cdot \sigma$$

where *ε* is the engineering strain, *λ* is the stretching ratio, and *σ* is the engineering stress, while *K*, *A*, and *B* are constants and could be obtained by fitting the stress–strain curve. When *σ* converges to 0, the elastic fibers are first stretched and then Equation (1) transforms into:

(2) $$E_{Elastic\;fibers}={\left.\frac{d\sigma }{d\varepsilon }\right|}_{\sigma \to 0}=\frac{1}{K+\frac{A}{B}}$$

At this stage, the elastic modulus should remain constant. As the strain gradually increases, the elastic fibers become fully stretched and the collagen fibers begin to bear the load. During this process, the stiffness of the aorta is determined by both collagen and elastic fibers, and Equation (2) transforms to:

(3) $$E_{collagenous\;fibers}+E_{Elastic\;fibers}={\left.\frac{d\sigma }{d\varepsilon }\right|}_{\sigma \gg B}=\frac{1}{K}$$

At this stage, the elastic modulus becomes constant again. Therefore, we can obtain the elastic modulus of elastic and collagen fibers, respectively.

#### 2.3.2. Energy Loss

The energy loss represents the energy dissipated by the aorta as blood flow kinetic energy converted to elastic potential energy during the cardiac cycle. A greater energy loss indicates more energy dissipated during aortic wall deformation, which may lead to aortic remodeling and failure [14]. Energy loss was found to be relatively insensitive to loading conditions and could reflect the compliance of the aortic wall [5]. As defined by Franchini et al. [22], the energy loss is specifically described as:

(4) $$\eta =\frac{\int_{cycle}\sigma d\varepsilon }{\pi \left(A_{1}+A_{2}\right)}$$

where *η* represents the energy loss factor, *ε* is the engineering strain, *σ* is the engineering stress, while *A*_1_ and *A*_2_ are the areas of the two curvilinear triangles in the stress–strain curve. It should be noted that *η* is a dimensionless parameter.

#### 2.3.3. Peeling Force

Peeling force is a direct reflection of the ability of the aortic wall to resist tearing. A lower peeling force represents a weaker ability of the aortic wall to resist tearing and a higher risk of AD development. The peeling force is defined as:

(5) $$PF=\frac{\int_{l}Fdl}{L}$$

where *PF* represents the average peeling force required to tear the aortic wall per unit length, *L* represents the peeling length, and *F* represents the force during the tearing process.

### 2.4. Statistical Analysis

All data processing was performed using SPSS Statistics, Version 18.0 (IBM Corp., New York, NY, USA), and Python 3 code developed by our team. Continuous variables were presented as mean ± standard deviation (SD). The Shapiro–Wilk test was used to assess the normality of the distribution. Differences among multiple groups were analyzed using one-way analysis of variance (ANOVA) for data that adhered to a normal distribution and the Kruskal–Wallis test for non-normally distributed data. The Tukey–Kramer test was used for post hoc pairwise comparisons, as it is suitable for data groups with unequal sample sizes. A *p*-value of less than 0.05 was considered statistically significant.

## 3. Results

The biomechanical characteristics of porcine aortic samples under stable and unstable hypertension conditions are presented in Table 1. Overall, various hypertension conditions significantly impact biomechanical characteristics of the porcine aortic wall.

### 3.1. Elastic Modulus under Different Hypertension Conditions

**Figure 2:**
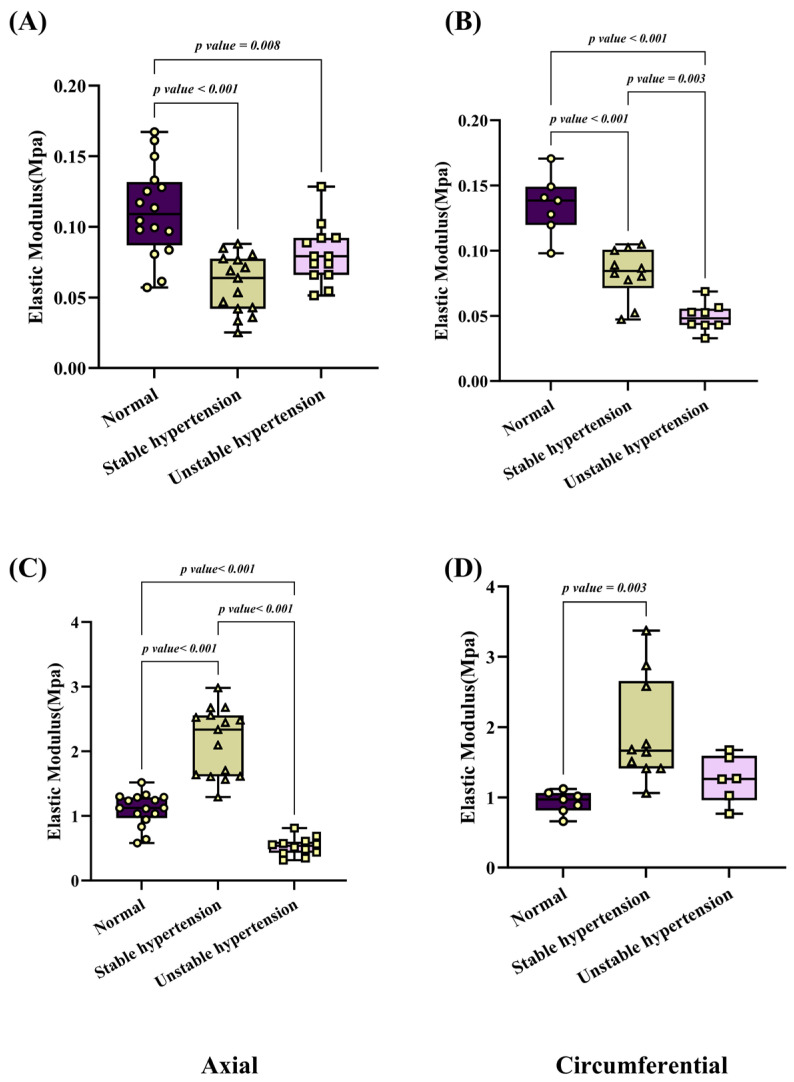
Elastic modulus of elastin and collagen fibers. (**A**) Modulus of elastin fiber in axial direction; (**B**) modulus of elastin fiber in circumferential direction; (**C**) modulus of collagen fiber in axial direction; (**D**) modulus of collagen fiber in circumferential direction.

**Figure 3:**
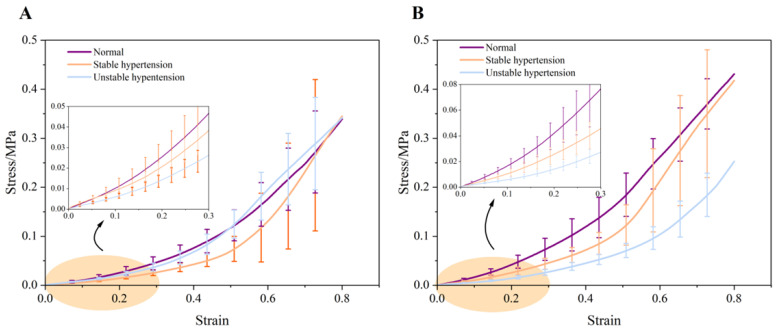
Average stress–strain curves for all porcine aortic wall samples in the (**A**) axial direction; (**B**) circumferential direction.

Figure 2 illustrates the changes in the elastic modulus of porcine aortas under different hypertension conditions. In the axial direction, the elastic modulus of elastic fibers under normal blood pressure is 0.11 ± 0.03 MPa. Under stable hypertension, the elastic modulus decreases to 0.05 ± 0.02 MPa (vs. normal blood pressure, *p* < 0.001). Under unstable hypertension, the decrease in elastic modulus is slightly less pronounced than the stable hypertension condition (0.08 ± 0.02 MPa compared to normal blood pressure, *p* = 0.008). For collagen fibers, the elastic modulus under normal blood pressure is 1.10 ± 0.24 MPa, while, under stable hypertension, the elastic modulus increases to 2.14 ± 0.51 MPa (vs. normal blood pressure, *p* < 0.001), while, under unstable hypertension, the elastic modulus of collagen fibers decreases significantly to 0.52 ± 0.14 MPa (vs. to normal blood pressure, *p* < 0.001). Notably, there is a turning point in the stress–strain curve at around 50% strain under unstable hypertension, where the elastic modulus gradually decreases and eventually falls below that of normal blood pressure. The magnified view at 30% strain shows that the elastic modulus under normal blood pressure is significantly higher than both stable and unstable hypertension conditions, as shown in Figure 3A.

In the circumferential direction, the elastic modulus of elastic fibers under normal blood pressure is 0.13 ± 0.02 MPa. After stable hypertension, it decreases to 0.08 ± 0.02 MPa (vs. normal blood pressure, *p* < 0.001) and further decreases to 0.05 ± 0.01 MPa under unstable hypertension (vs. normal blood pressure, *p* < 0.001). For collagen fibers, the elastic modulus under normal blood pressure is 0.93 ± 0.15 MPa, which increases to 1.93 ± 0.71 MPa under stable hypertension (vs. normal blood pressure, *p* = 0.003) and becomes 1.26 ± 0.31 MPa under unstable hypertension (vs. normal blood pressure, *p* = 0.53). However, statistical difference was not observed between normal blood pressure and unstable hypertension. The magnified view at 30% strain in the circumferential direction also shows consistent results, as presented in Figure 3B.

### 3.2. Energy Loss under Different Hypertension Conditions

**Figure 4:**
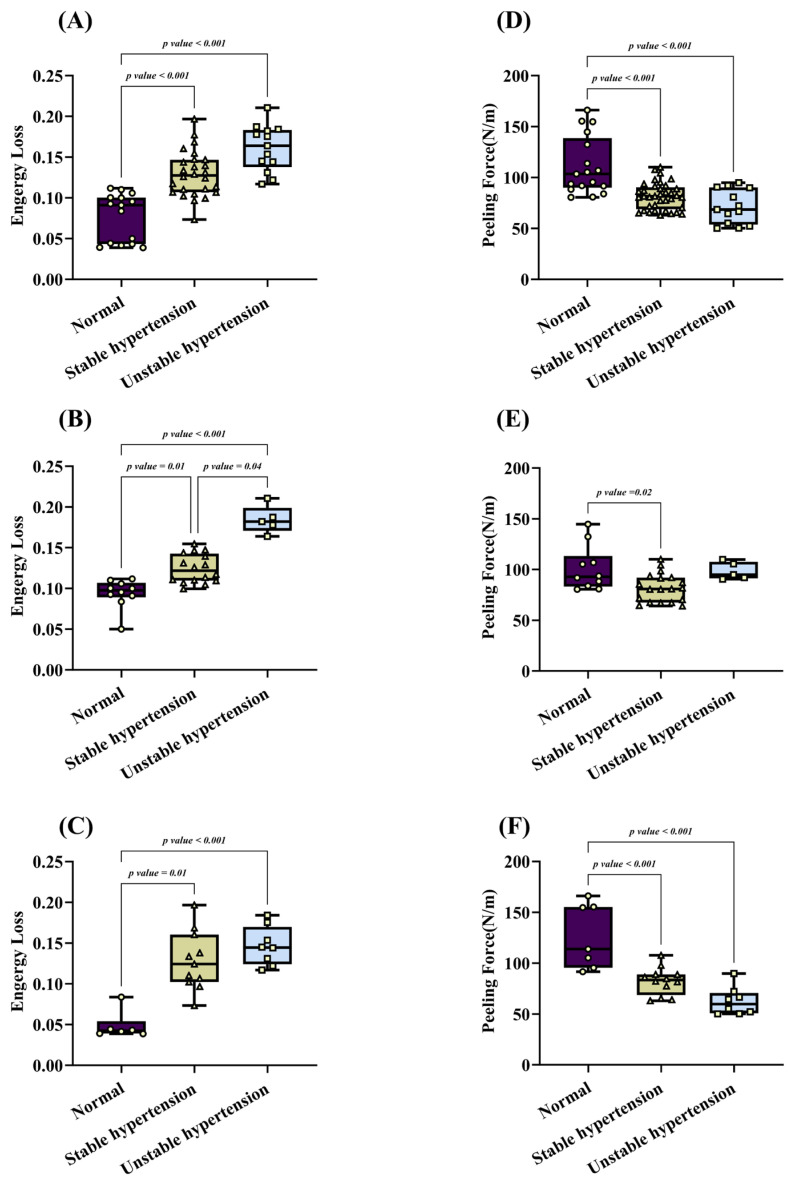
Energy loss and peeling force of porcine aortic wall samples. (**A**) Overall distribution of energy loss; (**B**) energy loss in axial direction; (**C**) energy loss in circumferential direction; (**D**) overall distribution of peeling forces; (**E**) peeling forces in axial direction; (**F**) peeling forces in circumferential direction.

The energy loss under different hypertension conditions is exhibited in Figure 4A–C. Under normal blood pressure, the overall energy loss is 0.08 ± 0.03, which increases significantly to 0.13 ± 0.03 under stable hypertension (vs. normal blood pressure, *p* < 0.001). Under unstable hypertension, the energy loss further increases to 0.16 ± 0.03 (vs. normal blood pressure, *p* < 0.001).

Given the anisotropic nature of the aortic wall, energy loss differs significantly in both axial and circumferential directions. In the circumferential direction, there is no statistical difference between stable and unstable hypertension conditions (0.13 ± 0.03 vs. 0.15 ± 0.02, *p* = 0.90), but both are significantly higher than the energy loss under normal blood pressure, as shown in Figure 4C. In the axial direction, unstable hypertension exhibits a very high energy loss of 0.18 ± 0.02, which is significantly greater than the energy loss under both stable hypertension (0.12 ± 0.02, *p* = 0.04) and normal blood pressure (0.09 ± 0.02, *p* < 0.001).

### 3.3. Peeling Force under Different Hypertension Conditions

Figure 4D–F demonstrate the peeling force under different hypertension conditions. Various hypertension conditions significantly affect the intramural tear resistance of aortas, with stable and unstable hypertension conditions notably reducing this resistance. Under normal blood pressure, the peeling force is 111.10 ± 27.65 N/m, which significantly decreases to 81.69 ± 12.72 N/m (*p* < 0.001) under stable hypertension and further decreases to 71.37 ± 16.13 N/m (*p* < 0.001) under unstable hypertension.

In the axial direction, the peeling force under normal blood pressure is 100.83 ± 20.89 N/m, which significantly decreases to 82.05 ± 13.49 N/m under stable hypertension (*p* = 0.02). Under unstable hypertension, the peeling force is 98.57 ± 7.68 N/m, showing no statistical difference compared to normal blood pressure (*p* = 0.02). In the circumferential direction, the trend is similar across the three groups: the highest peeling force is observed under normal blood pressure (126.03 ± 29.26 N/m), while it significantly decreases under both stable hypertension (82.38 ± 12.93 N/m) and unstable hypertension (62.61 ± 12.87 N/m).

## 4. Discussions

Hypertension is considered a significant risk factor for the development of TBAD. However, despite increasing awareness among clinicians and patients about the importance of blood pressure management, there is still insufficient recognition of the necessity for strict and regular blood pressure control. In this study, we assessed and quantified the alterations in the biomechanical properties of porcine aortic walls under both stable and unstable hypertension conditions, and the results show that both stable and unstable hypertension significantly impair the mechanical properties of the porcine aortic wall, with unstable hypertension causing relatively greater damage. This finding provides a theoretical basis for strict blood pressure management in hypertensive patients.

Stable and unstable hypertension both lead to a significant decrease in the elastic modulus of the elastic fibers, while the elastic modulus of the collagen fibers exhibited a substantial increase, particularly in the circumferential direction, which may be a result of mechanical damage on the collagen fibers. The aorta, as a viscoelastic material, experiences collagen fiber damage within the aortic wall after fatigue stretching and potentially makes the aortic wall stiffer, ultimately resulting in an increased elastic modulus. In fact, a study has also found that a severe damage zone already exists around the initial tear before the onset of AD [23].

The mechanical damage on the collagen fibers induced by the hypertension conditions may be an explanation for the increase in collagen content. Aortas in the human body are able to undergo a self-repair process; however, collagen fiber damage from stable or unstable hypertension may affect vascular remodeling, leading to an increase in collagen content within the aortic wall. Wang et al. has demonstrated that patients with TAD exhibited increased collagen content in the aortic wall, alongside significant apoptosis of smooth muscle cells (SMCs) [24]. This suggests that an imbalance in collagen fiber content, whether excessive or insufficient, can compromise the biomechanical properties of the aortic wall. Insufficient collagen fiber directly reduces wall stiffness, while excessive collagen accumulation leads to fibrosis, decreased arterial compliance, and obstructed blood supply to the aortic wall, causing SMC necrosis—factors that may contribute to AD. In fact, tissue samples from ascending aortic aneurysms have been reported to possess a significantly higher elastic modulus than the normal ascending aorta, and ascending aortic aneurysms at a higher risk of rupture also tend to exhibit a higher elastic modulus [13,25].

Compliance reflects the ability of an aorta to contract and expand, which is a crucial biomechanical indicator. Compliant aortas can store the blood pumped during systole by dilating the aortic wall and release it during diastole, ensuring a steady, continuous flow throughout the cardiac cycle. An in vitro study demonstrated that higher pressures reduce aortic wall compliance, while another study confirmed that a significant reduction in aortic compliance markedly increases systolic and pulse pressures [26,27]. Our findings further indicate that stable hypertension, particularly unstable hypertension, results in greater energy loss, indicating worse compliance. Greater energy loss indicates that more strain energy is absorbed by the aortic wall and, when the absorbed energy exceeds the wall’s capacity, damage to the aorta occurs, increasing the likelihood of material failure. This failure may manifest as collagen fiber rupture or the formation of an initial tear in the aortic wall. Under conditions of stable hypertension, especially unstable hypertension, the elasticity and compliance of the aortic wall decrease, where the reduced compliance, in turn, raises blood pressure, exacerbating the loss of elasticity and compliance in the aortic wall, thus creating a vicious cycle.

Intimal rupture is one of the initiating factors for the development of TBAD [1]. However, not all patients with intimal rupture progress to acute TBAD, and some may undergo spontaneous healing without evident symptoms [28]. This phenomenon may be attributed to the aortic wall’s inherent resistance to tearing. Under conditions of stable or unstable hypertension, the peeling forces, both circumferentially and axially, are significantly reduced. Consequently, when an initial rupture occurs, the aortic wall is more prone to interlaminar separation, ultimately leading to the development of TBAD.

The results of histological assessment, as illustrated in Supplementary Results (Supplementary Materials), further confirmed alterations in biomechanical properties. Elastic and collagen fibers are key components affecting the mechanical properties of the aortic wall, and our findings demonstrated notable degeneration of elastic and collagen fibers in the intima–media of the aortic wall, which may contribute to the deterioration of various biomechanical characteristics.

Overall, although both hypertension states can severely impair the mechanical properties of the aorta, our results further suggest that unstable hypertension may cause more significant damage compared to stable hypertension. In fact, it has been reported that inadequately managed hypertension and reduced patient compliance can lead to malignant increases in blood pressure, triggering hypertensive emergencies [29]. Therefore, it is crucial for hypertensive patients to strictly follow medical advice and maintain blood pressure within a reasonable range to prevent more severe damage to the aortic wall. Additionally, studies have noted that intense physical activities, such as weightlifting or intensive swimming, which cause a sudden sharp increase in blood pressure, can also lead to AAD in healthy individuals [30], suggesting that such rapid fluctuations in blood pressure can significantly harm the mechanical properties of the aortic wall, consistent with our findings. Consequently, individuals with high-risk anatomical factors for aortic dissection, such as a higher question mark degree, should avoid such activities to reduce the risk of AD [31,32]. Maintaining blood pressure within a reasonable range and avoiding drastic fluctuations are crucial for reducing the risk of TBAD and ensuring long-term health.

This study has several limitations. Firstly, this study simulates different hypertension states by controlling the strain rate. However, this approach does not precisely determine the corresponding physiological blood pressure values for the simulated hypertension states. Secondly, this study used ex vivo healthy porcine aortas for mechanical testing instead of human aortas. Most existing studies use aortic samples from diseased tissue, but the remodeling process caused by disease alters the biomechanical properties of the aorta, making it unclear whether observed differences are due to the experimental blood pressure conditions in the current study. Therefore, this study used healthy porcine aortas as an alternative. Thirdly, the aorta experiences complex multi-axial stress conditions in vivo, while the uniaxial tensile experimental approach employed in this study cannot comprehensively elucidate the directional variations in the mechanical properties of the aortic wall. Lastly, stable and unstable hypertension conditions cause significant tissue remodeling in the aortic wall, which this study cannot simulate.

## 5. Conclusions

Our study indicates that both stable and unstable hypertension significantly impair the biomechanical properties of the aortic wall, with unstable hypertension leading to greater biomechanical damage. Clinicians should adopt more stringent blood pressure management protocols for hypertensive patients, ensuring careful regulation of antihypertensive medications to prevent unstable blood pressure resulting from inappropriate drug use. Additionally, hypertensive patients must rigorously follow medical advice to maintain stable blood pressure, thereby avoiding unstable hypertension and further mechanical damage to the aorta, which can reduce the risk of AD. Future research could conduct in vivo experiments in animal models to explore the specific impacts of different hypertensive conditions on aortic wall remodeling and other risk factors of TBAD such as atherosclerosis, as well as the relationship between these factors and changes in mechanical performance.

## Figures and Tables

**Table 1:** Overall biomechanical characteristics in normotension, stable hypertension, and unstable hypertension.

	Normotension	Stable Hypertension	Unstable Hypertension	*p* Value
Biomechanical Characteristics				
Elastic Modulus (Mpa)				
Elastin fiber				
Axial	0.11 ± 0.03	0.05 ± 0.02	0.08 ± 0.02	<0.001
Circumferential	0.13 ± 0.02	0.08 ± 0.02	0.05 ± 0.01	<0.001
Collagen fiber				
Axial	1.10 ± 0.24	2.14 ± 0.51	0.52 ± 0.14	<0.001
Circumferential	0.93 ± 0.15	1.93 ± 0.71	1.26 ± 0.31	0.003
Energy Loss	0.08 ± 0.03	0.13 ± 0.03	0.16 ± 0.03	<0.001
Axial	0.09 ± 0.02	0.12 ± 0.02	0.18 ± 0.02	<0.001
Circumferential	0.04 ± 0.02	0.13 ± 0.03	0.15 ± 0.02	0.001
Peeling Force (N/m)	111.10 ± 27.65	81.69 ± 12.72	71.37 ± 16.13	<0.001
Axial	100.83 ± 20.89	82.05 ± 13.49	98.57 ± 7.68	0.01
Circumferential	126.03 ± 29.26	82.38 ± 12.93	62.61 ± 12.87	<0.001

Continuous variables are presented as mean ± standard deviation (SD).

## Data Availability

The original contributions presented in this study are included in the article and Supplementary Materials. Further inquiries can be directed to the corresponding authors.

## Supplementary Materials

The following supporting information can be downloaded at: https://www.mdpi.com/article/10.3390/biomedicines12102246/s1, Figure S1. Histological staining results under different blood pressure conditions. (A) Normal blood pressure; (B) Stable hypertension; (C) Unstable hypertension.
