# Supplementary material for: Hypertension-Induced Biomechanical Modifications in the Aortic Wall and Their Role in Stanford Type B Aortic Dissection
Source: Biomedicines. 2024 Oct 2;12(10):2246. doi: 10.3390/biomedicines12102246 (PMC11504259; doi:10.3390/biomedicines12102246)
Supplement: Supplementary file 1 [file biomedicines-12-02246-s001.zip › biomedicines-3202994-supplementary.pdf]

Supplementary Methods

Uniaxial Tensile Experiment

The cut aortic samples were secured in the clamp, with a rough-surfaced spacer placed on the clamp to prevent slipping. After clamping, a small displacement was applied to ensure the sample was taut. The length of the sample between the clamps was measured and recorded as the original length.

Researches indicate that the diameter of a healthy aorta typically varies by less than 10% during the cardiac cycle under normal blood pressure conditions [18,19]. In hypertensive conditions, the aortic wall experiences greater pressure, resulting in higher strain values than under normal blood pressure. To simulate the behavior of a healthy aorta under different blood pressure conditions, different strain rates were applied. A 10% strain rate was used to simulate normal blood pressure, while a 30% strain rate represented hypertension. After subjecting the samples to these conditions, their biomechanical properties were then assessed. The experimental procedures are as follows:

Normal Blood Pressure Fatigue Stretch Test

This test was conducted at a 10% strain rate with a fatigue cycle of 7200 cycles and a stretching frequency of 1 Hz, serving as the control group.

Hypertensive Fatigue Stretch Test

Here, the strain rate was set to 30%, with a fatigue cycle of 7200 cycles and a stretching frequency of 1 Hz. The experiment was conducted after ensuring no destructive stress during the fatigue stretch test.

Unstable Hypertensive Fatigue Stretch Test:

Initially, the strain rate was set to 30%, with a fatigue cycle of 1800 cycles and a stretching frequency of 1 Hz. After this phase, the strain rate was reset to 10%, while maintaining the same fatigue cycle and stretching frequency, and the experiment continued. This process was repeated once more. Ultimately, the samples underwent alternating fatigue stretching tests at strain rates of 30% and 10%, totaling 7200 cycles.

After completing the fatigue stretching tests, the biomechanical properties of porcine aortic walls were measured. The specific procedures and settings were as follows:

Energy Loss Test

The sample was fixed in a uniaxial stretching machine and stretched at a speed of 0.1 mm/s to a 30% strain, then returned to its original position at the same speed. This quasi-static stretching was repeated 10 times, and the loading-unloading curve from the 10th stretch was used to calculate the energy loss of the sample.

Peeling Force Test

Following the energy loss test, a 5 mm deep incision was made in the media of specimen using a scalpel. The torn parts of the specimen were then fixed on both sides of the uniaxial stretching machine and peeled to a length of 15 mm. The average peeling force was then calculated.

Elastic Modulus Test for Elastic and Collagen Fibers

After the fatigue test, the specimen was fixed in a uniaxial stretching machine and stretched at a speed of 0.1 mm/s until the material completely ruptured.

Histological Assessment

Paraffin sections with a thickness of 6μm were prepared from the processed porcine aortic samples. Five sections were taken from each sample. The tissue sections were then stained using hematoxylin and eosin (H&E staining). After staining, the sections were scanned using a digital pathology scanner (Pannoramic MIDI) and photographed. The morphology of the elastic and collagen fibers was subsequently evaluated.

Supplementary Results

Histological Assessment

Figure S1 shows the stained tissue sections of the aortic wall in the circumferential direction under different blood pressure conditions.

As illustrated in **Figure S1A**, under normal blood pressure, the elastic and collagen fibers within the aortic wall are tightly distributed at the intima-media junction and within the media. The fibers are arranged in an orderly, layered manner.

However, under stable hypertension condition (**Figure S1B**), the intima cells become loosely connected, and the elastic and collagen fibers at the intima-media junction become lax. The interweaving of fiber layers weakens, and the fibers become more crimped, indicating fiber rupture. Microcracks, approximately 0.1 mm in length, appear within the media, reducing the density of interwoven fiber layers.

Under unstable hypertension condition (**Figure S1C**), the elastic fibers in the medial region near the intima exhibit significant disordered crimping, and the boundary between the intima cells and the media almost disappears. Microcracks are also observed within the media, with some areas showing large cracks exceeding 0.5 mm in length.

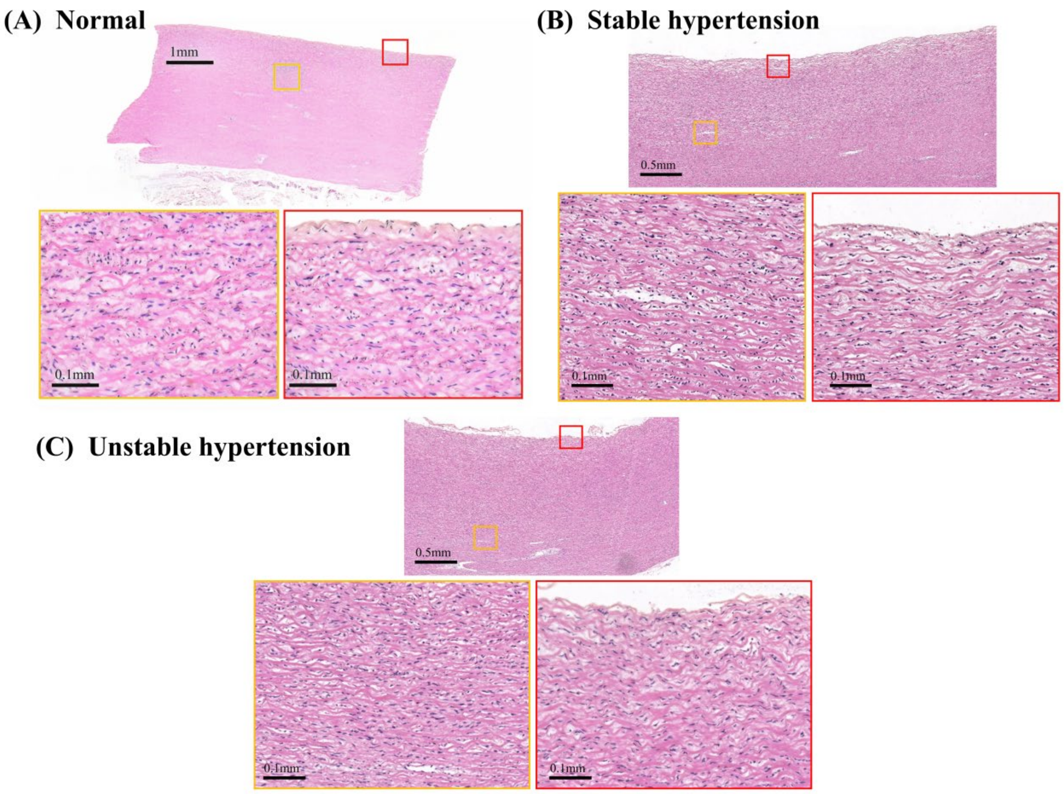

Figure S1. Histological staining results under different blood pressure conditions. (**A**) Normal blood pressure; (**B**) Stable hypertension; (**C**) Unstable hypertension.
